# The Influence of the Tri-reference Points on Fairness and Satisfaction Perception

**DOI:** 10.3389/fpsyg.2018.00193

**Published:** 2018-02-19

**Authors:** Lei Zhao, Junhui Ye, Xuexian Wu, Fengpei Hu

**Affiliations:** ^1^Center for Brain Science, College of Economics and Management, Zhejiang University of Technology, Hangzhou, China; ^2^Department of Social Science, Zhejiang Police College, Hangzhou, China; ^3^Zhejiang Institute of Communications, Hangzhou, China

**Keywords:** tri-reference point theory, fairness perception, satisfaction, social comparison, pay evaluation

## Abstract

We examined the influence of three reference points (minimum requirements [MR], the status quo [SQ], and goal [G]) proposed by the tri-reference point (TRP) theory on fairness and satisfaction perceptions of pay in three laboratory experiments. To test the effects, we manipulated these three reference points both implicitly (Experiment 1) and explicitly (Experiments 2 and 3). We also provided the information of the salary offered to a peer person that was lower than, equal to, or higher than the salary offer to the participant. As hypothesized, the results demonstrated the important role of these reference points in judging the fairness of and satisfaction with pay when they were explicitly set (an interaction between reference points and social comparison in Experiments 2 and 3, but not in Experiment 1). Participants altered their judgments when the salary was in different regions. When the salary was below MR, participants perceived very low fairness and satisfaction, even when the offer was equal to/exceeded others. When the salary was above G, participants perceived much higher fairness and satisfaction, even with disadvantageous inequality. Participants were more impacted when they were explicitly instructed of the reference points (Experiments 2 and 3) than when they were not (Experiment 1). Moreover, MR appeared to be the most important, followed by G. A Salary below MR was judged as very unacceptable, with very low fairness and satisfaction ratings.

## Introduction

It is widely accepted that the perception of fairness is of particular importance to compensation decisions, such as pay. Indeed, the matter of pay distribution plays an important role in shaping an employee’s attitude and behavior toward his work (Porter et al., 2003). Scholars have demonstrated that fairness perception has a large influence on organizationally relevant variables such as job satisfaction, organizational citizenship behaviors, organizational commitment, job performance, trust, absenteeism, and turnover (e.g., Cohen-Charash and Spector, 2001; Colquitt et al., 2001; Lahuis et al., 2007; Fassina et al., 2010). However, many employees feel that they are paid unfairly, leading to a critical need for greater understanding of how they perceive the fairness of their pay (Heneman and Judge, 2000).

All major theories assume that fairness perception is based on social comparisons. The fundamental model of such comparison theories is the equity theory proposed by Adams, which assumes that individuals make fairness judgments by comparing their rewards/contributions ratios to those of other individuals, termed referents (Adams, 1963a,b). Inequity occurs when the ratio is not equal to the referent’s ratio, being either overpaid (advantageous inequality) or underpaid (disadvantageous inequality). Goodman (1974) presented a conceptual framework containing three classes of referents for determining how individuals evaluate their pay. Subsequent studies based on Goodman’s work have attempted to investigate which referents are used in pay evaluation and found strong support for significant effects in five distinguishable categories: social, financial, historical, organization, and market. For example, Austin et al. (1980) found that social comparisons exhibit more importance in subjects’ judgments about the fairness of their pay. Messé and Watts (1983) further extended these findings by showing that internal standards, as well as social comparison, can influence fairness perception. Others have also documented the impact of expectations as reference points in the evaluation of pay (Cherry et al., 2003).

There is considerable evidence that the judged fairness of an outcome is significantly influenced by multiple reference points rather than a single one (e.g., Ordóñez et al., 2000). An increasing number of studies have also shown that people make decisions using multiple reference points. Wang and Johnson (2012) integrated research findings from behavior decision-making, management science, and risk-sensitive foraging theory and proposed a tri-reference point (TRP) theory to account for a serial of risk perception and risky choice behaviors. The TRP theory argues that the importance of the current state (or status quo [SQ]), the goal (G) and the survival-related minimum requirements (MR) reference points in determining people’s judgment and decision-making. The three reference points divide the outcome space into four functional regions: failure (below MR), loss (between MR and SQ), gain (between SQ and G) and success (equal or above G), and people have different risk cognitions and preferences in these functional regions. The authors tested the TRP theory empirically and showed that people consider both G and MR under a risky situation, switching between risk seeking and risk aversion when the outcome straddles a different reference point, which could not be explained by single reference point based models such as prospect theory.

According to TRP, when the mean expected value of an outcome is below MR (or G), people tend toward risk seeking to exceed the reference point. While the mean expected value is above MR (or G), people tend toward risk aversion to ensure that they reach the bottom line (goal). The psychological value of a same change in objective value is subjectively greater when it passes a reference point (e.g., from failure to loss) than when it remains in the same region. Additionally, the relative importance of these reference points differs, with an order of MR > G > SQ for their psychological impacts. The settings of these reference points are mainly determined by social and situational factors in task environments (e.g., economic, social, relational, and so on).

Researchers have applied the TRP theory in the organizational management field and have shown that these reference points indeed have an important influence on job related factors (turnover, job selecting). For example, Xiong et al. (2014) explore the relationship between salary gap and turnover regarding TRP. They found that the perceived salary gap between the SQ and MR of an employee negatively predicted the turnover intention; the gap between G and SQ positively predicted the turnover intention. Wang and Sang (2009) examined the influence of TRP on job selection of different types of salary offerings regarding college graduates. They conducted three types of salary offerings (fixed, high-variance, and low-variance) based on the estimated salary MR and G derived from the same sample population. The results revealed that the choice preference pattern of these college participants was consistent with the TRP theory: participants tended toward risk-seeking, preferred a high-variance salary job when the fixed salary was below MR and high-variance salary exceeded the MR with chance; when the fixed salary was above MR, participants tended toward risk-avoiding, with fewer participants choosing the high-variance salary job. The authors also explored the tradeoffs between MR and G and found that the participants gave priority to MR over G when the range of the salary variation extended beyond both reference points.

Perceptions of (un)fairness severely impact job related factors such as turnover and job selection. Unfair pay is an important reason given by employees for leaving their jobs. The influence of TRP on these factors may result from altering the perceived fairness. For example, Falk et al. (2006) found that temporary introduction of a minimum wage leads to a rise in subjects’ reservation wages which persists even after the removal of the minimum wage. Participants behaved as if they perceived less fairness after the introduction of the minimum wage. Other related works have also shown the importance of the MR in organizations. Giessner and van Knippenberg (2008) found that failing MR goals (but not maximal goal or G) resulted in equally negative evaluations of a leader independent of some additional information about this leader (how representative he/she is for the team). In the study reported here, we examine the influence of the TRP on the pay evaluation. Specially, to examine whether the reference points of MR, SQ, and G alter people’s perceived fairness. Since job satisfaction is also an important factor in organizational management and is highly related to fairness, we also tested this factor. A primary interest is how these reference points interact with the basis of fairness judgments (social comparison though the salaries received by similar others, e.g., Dornstein, 1989). In light of the above discussions, we would expect the TRP to have a significant influence on the perception of fairness of and satisfaction with a job salary. We are proposing the following hypothesis:

H1 The TRP will have a significant influence on the perception of fairness and satisfaction.

Specially, we formulated the following two sub-hypothesis:

H1a A salary below MR will be perceived as more unfair and the person will be dissatisfied, even with equality or advantageous inequality.
H1b A salary above G will be perceived as fairer and the person will be more satisfied regardless of the other’s pay.

Of course, though the concepts of fairness and satisfaction are related, they are distinct from each other and are likely to be affected by different reference points. The main difference occurs in the advantageous inequality. Typically, we are more satisfied with a higher salary, but we do not consider it to be fairer than equal salaries. Additionally, we are more likely to be unsatisfied/satisfied with low/high pay, we would also expect that MR/G would have greater influence on satisfaction than on fairness perception. Thus, we are proposing the following hypothesis:

H2 TRP will have different influences on fairness and satisfaction perception. The MR and G will have greater influence on satisfaction than fairness perception.

## Experiment 1

### Methods

According to Wang and Johnson (2012), the three reference points can be either determined endogenously or set exogenously (e.g., natural situation, task requirements, or social comparisons). Following their study, we also started with a pre-test to estimate the mean values of the three reference points and design the salary levels around these means. This allowed us to test our hypothesis in an endogenous way (without inducing the reference points to participants). During the pre-test, we surveyed 60 students from Zhejiang University of Technology (located in Hangzhou). The participants were asked to report their minimum required first-job salary for living in Hangzhou, their average expected (most likely) first salary, and their desired salary in Hangzhou. The roundup averages were 2800, 4600, and 6500 RMB per mouth. These three numbers were used as the MR, SQ, and G, respectively.

We then recruited 32 students from the same university to participate in our formal experiment with payment (RMB 5 for each participant). Similar to Ordóñez et al. (2000), participants were asked to respond to scenarios involving themselves as a graduate who has received a job offer (see “Supplementary Material” for details). Along with their own salary, participants were told about the salary of one similar graduating student (with the same gender, skill, education background, and so on) who had also received the offer. For each scenario, participants were instructed to respond to the questions, “How fair do you think the job offer is?” and, “How satisfied are you about the job offer?” on a seven-point scale (1: totally unfair [unsatisfied], 7: totally fair [satisfied]). Twelve scenarios were created using a 4 (salary levels) ^∗^ 3 (comparison with the other) within-subject design. The four levels of salary were 2200, 3700, 5100, and 7400 RMB that were located in the four regions (failure, loss, gain, and success) divided by the tri-reference points MR, SQ, and G. The salary of the other person was higher than (disadvantageous inequality), equal to, or lower than (advantageous inequality) the participant with the constraint that it was in the same regions as the participant (the absolute difference was randomly assigned between 200 and 400 in inequality conditions). There were four trials for each scenario. The order of the scenarios was randomized across participants. The total experiment took approximately 10 min.

#### Ethics Statement

All participants provided written informed consent before participating in the experiments. The participants were reminded of their right to discontinue participation at any time. The Research Ethics Board of Zhejiang University of Technology approved all procedures.

### Results and Discussion

#### Fairness Perception

**Table 1** summarizes the fairness perception in Experiment 1. A repeated-measures analysis of variance (ANOVA) was performed. Salary levels and comparison with the other were included as within-subject variables. The results indicated a significant main effect of the salary levels, *F*(3,93) = 24.387, *p* < 0.001, $\eta_{p}^{2}$= 0.440. *Post hoc* (Bonferroni) analysis showed that there were significant differences in fairness perception between all pairs of two regions (*M* = 4.31, 4.78, 5.06, and 5.42 for failure, loss, gain, and success, respectively, with all *p*s < 0.001 except for the failure/loss and loss/gain comparison, which showed *p* < 0.01 and *p* = 0.01, respectively). Perceived fairness was lowest when the salary was below the MR. However, when the salary was above G, participants perceived much higher fairness. This suggests that the tri-reference points indeed affect the perception of fairness. The main effect of comparison with the other was also significant, *F*(2,62) = 38.065, *p* < 0.001, $\eta_{p}^{2}$= 0.551. *Post hoc* (Bonferroni) analysis showed that an equal salary offer was judged as most fair (*M* = 5.76) compared to the other conditions (advantageous inequality: *M* = 4.67, disadvantageous inequality: *M* = 4.25, all *p*s < 0.001). The advantageous inequality condition was also judged as fairer than the disadvantageous inequality condition (*p* < 0.05). This was consistent with the equity theory. However, the interaction between the two variables was not significant, *F*(6,186) = 1.456, *p* = 0.222, $\eta_{p}^{2}$= 0.045, H1 was partially supported.

**Table 1:** Perceived fairness of Experiment 1 (mean rating with SD).

	Advantageous inequality	Equality	Disadvantageous inequality
Failure	4.09 (1.21)	5.29 (1.58)	3.55 (1.26)
Loss	4.63 (1.12)	5.65 (1.30)	4.06 (1.09)
Gain	4.80 (1.15)	5.91 (1.08)	4.46 (1.04)
Success	5.17 (1.10)	6.17 (1.01)	4.91 (1.05)

#### Satisfaction Perception

**Table 2** summarizes the satisfaction perception in Experiment 1. A repeated-measures ANOVA indicated a significant main effect of the salary levels, *F*(3,93) = 115.257, *p* < 0.001, $\eta_{p}^{2}$ = 0.788. *Post hoc* (Bonferroni) analysis showed that there were significant differences in fairness perception between all pairs of two regions (*M* = 2.89, 4.05, 4.94, and 5.78 for failure, loss, gain, and success, respectively, with all *p*s < 0.001). Perceived satisfaction was lowest when the salary was below the MR. However, when the salary was above G, participants perceived much higher satisfaction. This suggests the Tri-reference points affect the satisfaction perception as well as fairness. The main effect of comparison with the other was also significant, *F*(2,62) = 5.911, *p* < 0.05, $\eta_{p}^{2}$= 0.160. *Post hoc* (Bonferroni) analysis showed that the disadvantageous inequality condition was judged as leading to less satisfaction (*M* = 4.20) compared to the other conditions (advantageous inequality: *M* = 4.47, *p* < 0.01, equality: *M* = 4.57, *p* < 0.05). The satisfaction perception of advantageous inequality and equality condition was non-significant (*p* = 0.957). This was consistent with the idea that people may not see advantageous inequality as fairer than equal salaries but consider it as just satisfying their needs. Again, the interaction between the two variables was non-significant, *F*(6,186) = 0.966, *p* = 0.433, $\eta_{p}^{2}$ = 0.030, partially supporting H1.

**Table 2:** Perceived satisfaction of Experiment 1 (mean rating with SD).

	Advantageous inequality	Equality	Disadvantageous inequality
Failure	2.88 (1.12)	3.02 (1.36)	2.76 (1.09)
Loss	4.16 (1.10)	4.16 (1.31)	3.82 (1.07)
Gain	4.96 (0.97)	5.12 (1.06)	4.73 (1.15)
Success	5.86 (0.90)	5.98 (0.91)	5.50 (1.07)

## Experiment 2

Experiment 1 demonstrated that the TRP affects the perception of fairness and satisfaction with the implicit, self-determined reference points without imposing explicit reference points. However, the results failed to find an interaction between salary levels and comparison with the other, and thus did not fully support our hypothesis. This may be because implicit salary reference points were not salient enough to forcefully alter participants’ perception. Thus, in Experiment 2, we set these references exogenously to further explore the role of TRP in pay evaluation.

### Methods

The procedure was exactly the same as that in Experiment 1 except as noted below. Thirty naïve participants were tested. The tri-reference points were now set exogenously. Participants were informed about the minimum first-job salary for living in Hangzhou (MR, 2800 RMB), the average first salary (SQ, 4600 RMB), and the desired salary (G, 6500 RMB) of the same university students before completing the judgment task.

### Results and Discussion

#### Fairness Perception

**Table 3** summarizes the fairness perception in Experiment 2. There was a significant main effect of salary levels, *F*(3,87) = 65.395, *p* < 0.001, $\eta_{p}^{2}$ = 0.693. *Post hoc* (Bonferroni) analysis showed that there were significant differences in fairness perception between all pairs of two regions (*M* = 3.62, 4.44, 5.14, and 5.82 for failure, loss, gain, and success, respectively, with all *p*s < 0.001), just as in Experiment 1. Perceived fairness was lowest when the salary was below the MR. However, when the salary was above G, participants perceived much higher fairness, even with the disadvantageous inequality. The main effect of comparison with the other was also significant, *F*(2,58) = 26.417, *p* < 0.001, $\eta_{p}^{2}$ = 0.477. *Post hoc* (Bonferroni) analysis showed that an equal salary offer was judged as most fair (*M* = 5.44) compared to the other conditions (advantageous inequality: *M* = 4.63, disadvantageous inequality: *M* = 4.20, all *p*s < 0.001). The advantageous inequality was also judged as fairer than disadvantageous inequality (*p* < 0.01). This, again, was consistent with the equity theory. Different from Experiment 1, the interaction between the two variances was now significant, *F*(6,174) = 2.722, *p* < 0.05, $\eta_{p}^{2}$ = 0.086. *Post hoc* (Bonferroni) analysis showed that when below the MR, the advantageous and disadvantageous inequality conditions were not different from each other, suggesting that people perceived low fairness when their salary was in the failure region. The difference between all other pairs of conditions in other regions (loss, gain, and success) was significant. The interaction effect suggests that the outcome effect alone cannot explain the perceived fairness, especially in the failure regions. The results supported H1a and H1b.

**Table 3:** Perceived fairness of Experiment 2 (mean rating with SD).

	Advantageous inequality	Equality	Disadvantageous inequality
Failure	3.33 (1.28)	4.47 (1.78)	3.06 (1.16)
Loss	4.41 (0.91)	5.18 (1.41)	3.76 (0.99)
Gain	5.06 (0.68)	5.75 (0.90)	4.63 (1.05)
Success	5.73 (0.81)	6.38 (0.66)	5.35 (0.83)

#### Satisfaction Perception

**Table 4** summarizes the satisfaction perception in Experiment 2. A repeated-measures ANOVA indicated a significant main effect of the salary levels, *F*(3,87) = 346.881, *p* < 0.001, $\eta_{p}^{2}$ = 0.923. *Post hoc* (Bonferroni) analysis showed that there were significant differences in satisfaction perception between all pairs of two regions (*M* = 2.13, 3.55, 4.96, and 6.13 for failure, loss, gain, and success, respectively, with all *p*s < 0.001). Perceived satisfaction was lowest when the salary was below the MR. However, when the salary was above G, participants perceived much higher satisfaction. This also suggests that the tri-reference points affect the satisfaction perception as well as fairness. The main effect of comparison with the other was also significant, *F*(2,58) = 5.652, *p* < 0.01, $\eta_{p}^{2}$ = 0.163. *Post hoc* (Bonferroni) analysis showed that the disadvantageous inequality condition was judged as leading to less satisfaction (*M* = 4.06) compared to the other conditions (advantageous inequality: *M* = 4.26, *p* = 0.055, equality: *M* = 4.26, *p* < 0.05). The satisfaction perception of the advantageous inequality and equality condition was non-significant (*p* = 1.00). This was consistent with Experiment 1. Again, in contrast to experiment 1, the interaction between the two variances was significant, *F*(6,174) = 2.605, *p* < 0.05, $\eta_{p}^{2}$ = 0.082. However, different from fairness perception, *post hoc* (Bonferroni) analysis showed very different patterns for the satisfaction perception: there were no significant differences between any pairs of two conditions when the salaries were below the MR, suggesting that people always exhibit very low satisfaction when their pay was below the bottom level even with equal pay. The satisfactions of pay between any pairs of two conditions in the loss and gain regions were also undifferentiated. The only significant occurs in the success region. Participants felt less satisfied in the disadvantageous inequality condition than in the advantageous inequality (*p* < 0.01) or the equality conditions (*p* = 0.001). The difference between advantageous inequality condition and equality condition were non-significant (*p* = 0.185). These results supported H1a and H1b. The difference pattern of the satisfaction perception also supported H2.

**Table 4:** Perceived satisfaction of Experiment 2 (mean rating with SD).

	Advantageous inequality	Equality	Disadvantageous inequality
Failure	2.18 (0.91)	2.08 (0.77)	2.15 (0.79)
Loss	3.66 (0.85)	3.59 (0.84)	3.41 (0.91)
Gain	4.99 (0.63)	5.07 (0.55)	4.82 (0.71)
Success	6.22 (0.58)	6.31 (0.56)	5.86 (0.64)

## Experiment 3

Experiment 2 demonstrated that when participants were explicitly aware of the reference points, the evaluation of the pay was strongly affected. The interaction between salary levels and comparison with the other highly supported our hypotheses. However, the relative small sample size undermined the results. Thus, to test the robustness of our results, in Experiment 3, we replicated Experiment 2 with a larger group of participants.

### Methods

The procedure was exactly the same as that in Experiment 2 except as noted below. Based on the fairness interaction effect of our Experiment 2, we performed a power analysis with power of 0.95 in G^∗^power 3 (Faul et al., 2007) to calculate the sample size. The result suggested that we should get approximately 60 individuals to achieve the predicted effect size. Thus, sixty naïve participants were tested; the data from one participant was excluded due to being incomplete.

### Results and Discussion

#### Fairness Perception

**Table 5** summarizes the fairness perception in Experiment 3. The results were almost the same as that in Experiment 2. There was a significant main effect of salary levels, *F*(3,174) = 88.542, *p* < 0.001, $\eta_{p}^{2}$ = 0.604. *Post hoc* (Bonferroni) analysis showed that there were significant differences in fairness perception between all pairs of two regions (*M* = 3.65, 4.27, 4.83, and 5.33 for failure, loss, gain, and success, respectively, with all *p*s < 0.001), just as in Experiment 2. The main effect of comparison with the other was also significant, *F*(2,116) = 55.871, *p* < 0.001, $\eta_{p}^{2}$ = 0.491. *Post hoc* (Bonferroni) analysis showed that an equal salary offer was judged as most fair (*M* = 5.49) compared to the other conditions (advantageous inequality: *M* = 4.33, disadvantageous inequality: *M* = 3.73, all *p*s < 0.001). The advantageous inequality was also judged as fairer than disadvantageous inequality (*p* < 0.001). This, again, was consistent with the equity theory. The interaction between the two variances was also significant, *F*(6,348) = 3.015, *p* < 0.05, $\eta_{p}^{2}$ = 0.049. *Post hoc* (Bonferroni) analysis showed the same patterns as that in Experiment 2: when below the MR, the advantageous and disadvantageous inequality conditions were not different from each other, suggesting that people perceived low fairness when their salary was in the failure region. The difference between all other pairs of conditions in other regions (loss, gain, and success) was significant. The results again supported H1a and H1b.

**Table 5:** Perceived fairness of Experiment 3 (mean rating with SD).

	Advantageous inequality	Equality	Disadvantageous inequality
Failure	3.30 (1.40)	4.76 (1.65)	2.88 (1.14)
Loss	4.14 (0.97)	5.25 (1.27)	3.42 (1.00)
Gain	4.72 (1.08)	5.75 (1.10)	4.03 (1.04)
Success	5.19 (1.21)	6.20 (0.93)	4.60 (1.37)

#### Satisfaction Perception

**Table 6** summarizes the satisfaction perception in Experiment 3. The results were also almost the same as that in Experiment 2. A repeated-measures ANOVA indicated a significant main effect of the salary levels, *F*(3,174) = 642.110, *p* < 0.001, $\eta_{p}^{2}$= 0.923. Same as in Experiment 2, *Post hoc* (Bonferroni) analysis also showed that there were significant differences in satisfaction perception between all pairs of two regions (*M* = 1.79, 3.18, 4.76, and 6.17 for failure, loss, gain, and success, respectively, with all *p*s < 0.001). Perceived satisfaction was lowest when the salary was below the MR. However, when the salary was above G, participants perceived much higher satisfaction. The main effect of comparison with the other was also significant, *F*(2,116) = 10.619, *p* < 0.001, $\eta_{p}^{2}$ = 0.155. *Post hoc* (Bonferroni) analysis showed that the disadvantageous inequality condition was judged as leading to less satisfaction (*M* = 3.84) compared to the other conditions (advantageous inequality: *M* = 4.05, *p* < 0.001, equality: *M* = 4.03, *p* < 0.01). The satisfaction perception of the advantageous inequality and equality condition was non-significant (*p* = 1.00). This was consistent with Experiments 1 and 2. Again, the interaction between the two variances was significant, *F*(6,174) = 4.917, *p* < 0.001, $\eta_{p}^{2}$ = 0.078. Similar to Experiment 2, we also found very different patterns for satisfaction perception. *Post hoc* (Bonferroni) analysis showed that there were no significant differences between any pairs of two conditions when the salaries were below the MR, suggesting that people always exhibit very low satisfaction when their pay was below the bottom level even with equal pay. When the salaries were above G, participants felt less satisfied in the disadvantageous inequality condition than in the advantageous inequality or equality conditions (both *p*s < 0.001). The differences between the advantageous inequality condition and the equality condition were non-significant (*p* = 1.00), which supported H1a and H1b. The pattern in the loss region was similar to the failure region (the only significant difference occurred between the disadvantageous inequality and advantageous inequality conditions), and the pattern in the gain region was the same as in the success regions, consistent with our predictions. The difference pattern of the satisfaction perception again supported H2.

**Table 6:** Perceived satisfaction of Experiment 3 (mean rating with SD).

	Advantageous inequality	Equality	Disadvantageous inequality
Failure	1.75 (0.85)	1.79 (0.77)	1.82 (0.79)
Loss	3.29 (0.85)	3.20 (0.80)	3.04 (0.83)
Gain	4.87 (0.87)	4.83 (0.89)	4.59 (0.89)
Success	6.29 (0.68)	6.31 (0.72)	5.92 (0.89)

## General Discussion

A central question in our study was whether TRP involves in decision makers’ judgment about satisfaction and fairness of pay. The evidence suggests an influence of these references points, at least in the context of our stylized salary offer comparisons. This was not dependent on whether the reference point were set endogenously or exogenously; participants appeared to alter their judgments when the salaries were in different regions. Specially, when the salary was below MR, participants perceived very low fairness and satisfaction, even when the offer was equal to/exceeded others’ offer, especially for the exogenously setting. When the salary was above G participants perceived much higher fairness and had higher satisfaction, even with the disadvantageous inequality. Of course, the impact was greater when the participants were explicitly aware of the reference points than implicitly. The lack of interaction effects in Experiment 1 suggest that implicit suggestions may not salient enough to forcefully alter participants’ perception of fairness and satisfaction (though the pattern of the evaluation in each region was almost the same as that in Experiments 2 and 3). However, when they were aware of these reference points, the evaluation of the pay was strongly affected.

As predicted, fairness and satisfaction respond differently. Satisfaction was more influenced by TRP, especially by MR. People exhibited very low satisfaction when their pay was below the bottom level, whereas an equal salary was still judged as fairer than both types of inequality even in the failure region. Moreover, consistent with previous studies (Messick and Sentis, 1983), the asymmetry effects of advantageous/disadvantageous inequality in fairness and satisfaction were also supported in our study: disadvantageous inequality decreases satisfaction whereas advantageous inequality increases it (undifferentiated to equality in our study). However, participants judged both types of inequality as less fair than equality, with disadvantageous inequality as more unfair than advantageous inequality.

Previous studies have demonstrated the existence of the TRP in decision making under risk, as well as in other situations (e.g., turnover and job selection). Our study further supports this theory and extends it to the pay evaluation field. In line with Wang and Johnson (2012), our study also supports the relative importance of the psychological impacts of these reference points: MR was most important as a salary below MR was extremely unacceptable (with very low fairness and satisfaction ratings), even with equality (or advantageous inequality, for a satisfaction judgment); followed by the G as a salary achievement goals were judged as more fair and satisfied even with some inequality. This is also consistent with a longstanding security-first principle in financial and business management (e.g., Roy, 1952). The different effects of MR and G also suggest that the MR effects were less biased by peer comparison. This is also consistent with a previous finding (Hill and Buss, 2010) that risky choice is significantly affected by social comparison in gain situations (closer to a G), but not in loss situations (closer to the MR).

One might argue that the results of our study simply reflect the outcome effect; that is, people just feel fairer and more satisfied as their pay increases, there are no reference points involved in this situation. We highly doubt this as theoretical and practical evidence has suggested that the perceived fairness and satisfaction are mainly dependent on comparison (e.g., social comparison, internal standards) rather than the absolute outcome. More importantly, the different results of Experiment 1 and Experiments 2 and 3 (with no interaction in Experiment 1 and a significant interaction in Experiments 2 and 3) clearly showed the influence of these reference points. This is because the only difference between these two experiments was whether these reference points were mentioned before the judge task. Participants actually received the same salaries in these two experiments and were told the outcome of each scenario. If the only cause was the outcome effect, we would expect the same result patterns in both experiments. Clearly, when the reference points were not set explicitly, participants were less affected (as shown in Experiment 1), and when the participants explicitly know these reference points, the interaction effects occur for both fairness and satisfaction perception (as shown in Experiments 2 and 3). In addition, the results of Experiments 2 and 3 (opposite direction of evaluated ratings in the failure and success regions that participants tend to lower ratings below MR and increase ratings above G regardless of others’ pay [especially for the MR], and that the loss and gain regions were less impacted) were consistent with the TRP theory prediction and the proposal of the relative psychological impact importance of these reference points (MR > G > SQ). The outcome effect alone could hardly explain the pattern of the judged fairness and satisfaction observed in our study. Of course, further studies that directly manipulate the salary level and reference points (e.g., the same salary is evaluated when it is above or below a reference point, or different salaries within the same region are evaluated) are needed to better verify our results.

A potential limitation of the present research is that the scenario procedure we used in our experiments did not tap into participants’ actual experiences but rather their intuitions of what those experiences might be. The relationship between the actual experience and imaginary ones, clearly, is an important issue that needs to be addressed further. However, accurate or not, the participants’ anticipations of their reactions are of interest in their own right to the extent that they guide their judgments. For example, one may accept a job just because he/she anticipates a high fairness and satisfaction for the salary offered, even if the anticipations may not correctly reflect the actual experiences. Clearly, both anticipations and actual experiences are of import.

How individual judges experience relative fairness and satisfaction is of particular import for organizational management. Researchers have made efforts to explore the frames of reference guiding the judgments of individuals’ evaluation of payment and have achieved remarkable results (e.g., internal standards, social comparison, and expectations). By applying the new theoretically developed TRP theory in the field of pay evaluation, our study gives insights for the existing models and for practical implications. Results from our study suggest that, explicit or not, the bottom level and goal would significantly impact individuals’ perception of fairness and satisfaction about their pay. Fairness perception is not only based on some invariant moral standards, but is also judged against task-specific variables of goals and minimum requirements. Organizations should pay more attention to avoid falling below a bottom level and might consider how to manipulate the goal appropriately while designing compensation systems most likely to attract, motivate, and retain the best talent.

## Author Contributions

Conceived and designed the experiments: LZ, JY, XW, and FH. Performed the experiments: LZ. Analyzed the data: LZ, JY, and XW. Wrote the manuscript: LZ, JY, XW, and FH. All authors edited and approved the manuscript.

## Conflict of Interest Statement

The authors declare that the research was conducted in the absence of any commercial or financial relationships that could be construed as a potential conflict of interest.
