# Supplementary material for: The Influence of the Tri-reference Points on Fairness and Satisfaction Perception
Source: Front Psychol. 2018 Feb 19;9:193. doi: 10.3389/fpsyg.2018.00193 (PMC5825989; doi:10.3389/fpsyg.2018.00193)
Supplement: Supplementary file 1 [file Presentation_1.pptx]

## Slide 1
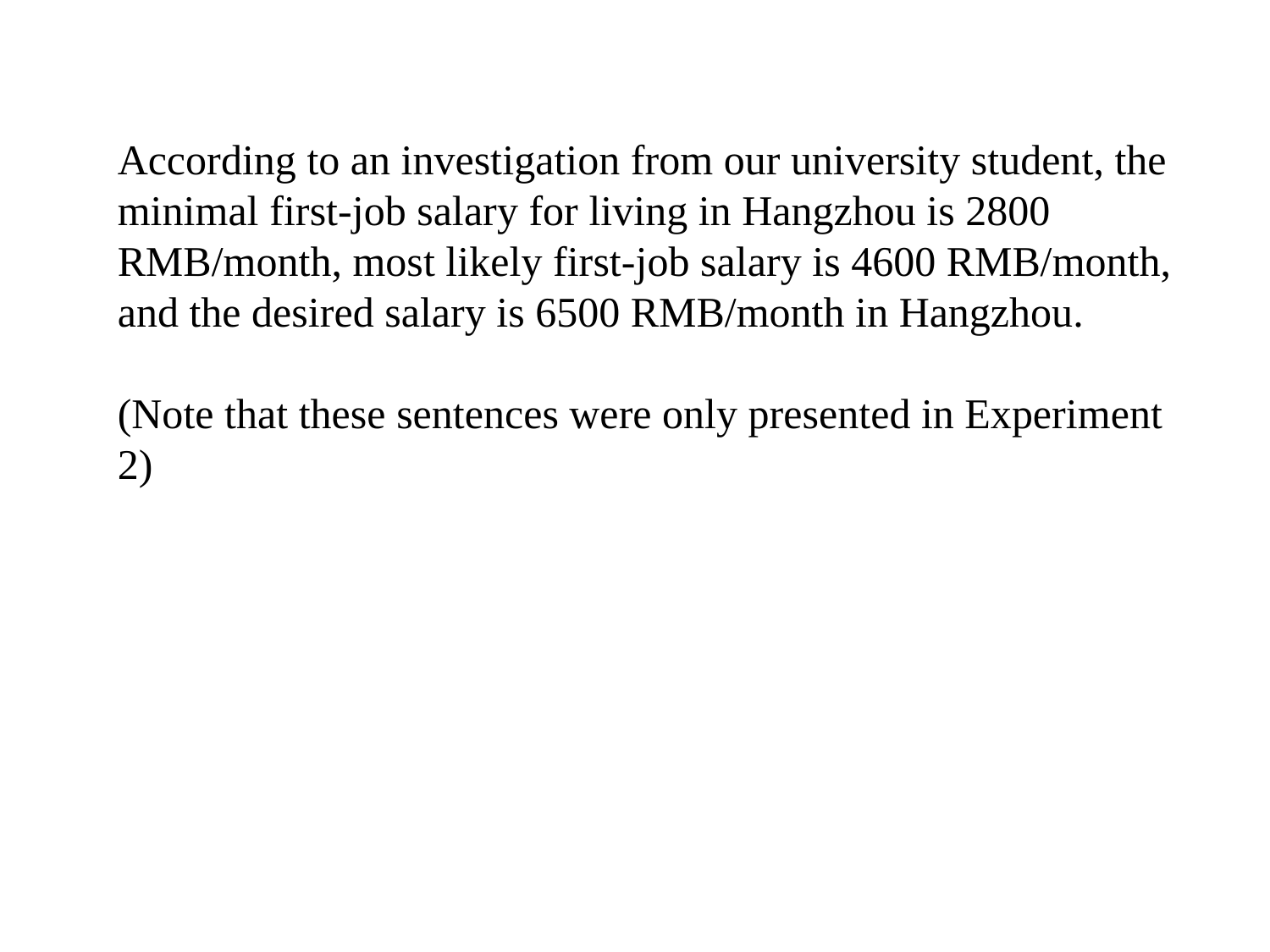

According to an investigation from our university student, the minimal first-job salary for living in Hangzhou is 2800 RMB/month, most likely first-job salary is 4600 RMB/month, and the desired salary is 6500 RMB/month in Hangzhou.
(Note that these sentences were only presented in Experiment 2)

## Slide 2
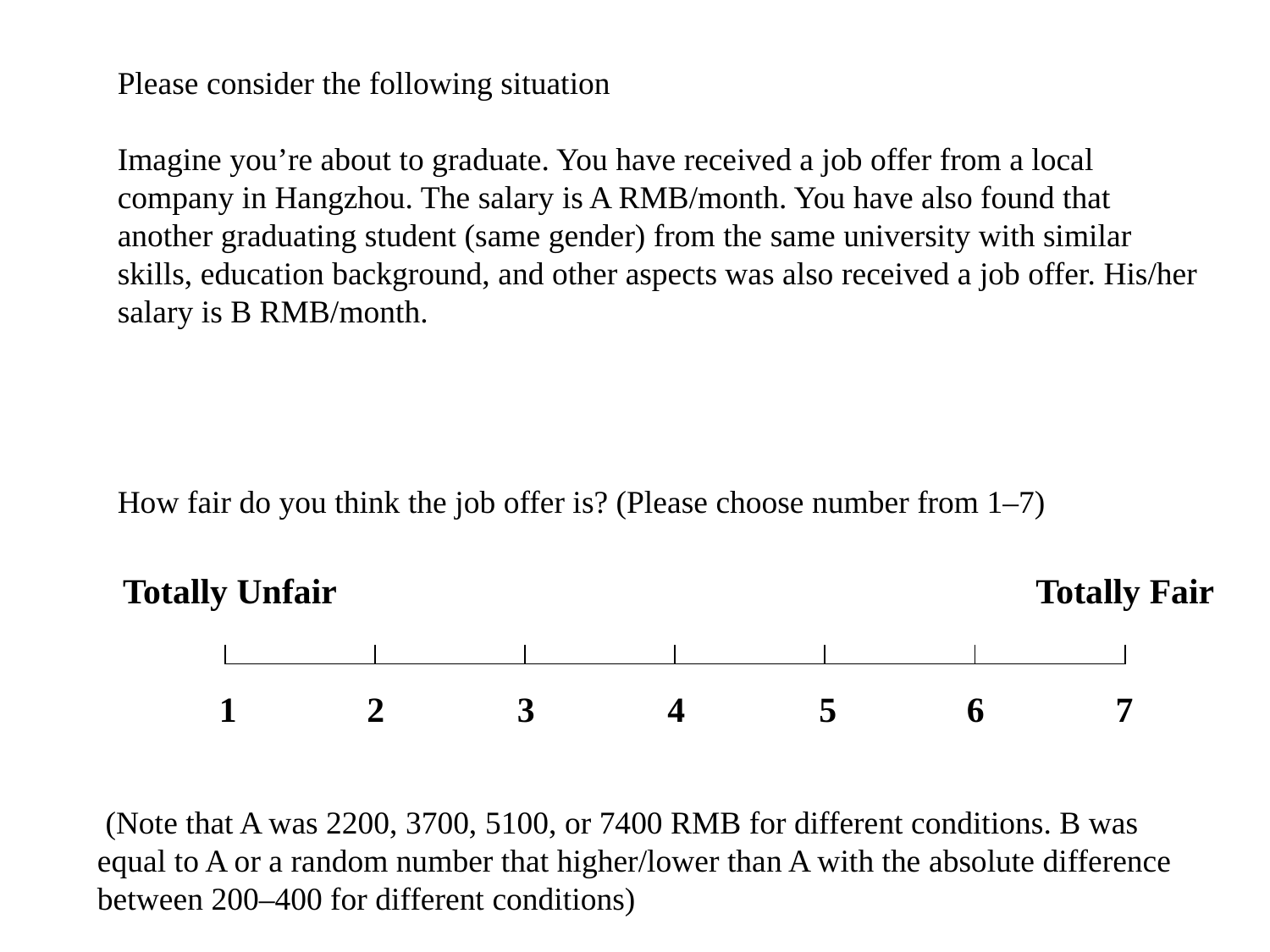

Please consider the following situation
Imagine you’re about to graduate. You have received a job offer from a local company in Hangzhou. The salary is A RMB/month. You have also found that another graduating student (same gender) from the same university with similar skills, education background, and other aspects was also received a job offer. His/her salary is B RMB/month.
How fair do you think the job offer is? (Please choose number from 1–7)
| | | | | | |
| --- | --- | --- | --- | --- | --- |
Totally Unfair
Totally Fair
1
2
3
4
5
6
7
 (Note that A was 2200, 3700, 5100, or 7400 RMB for different conditions. B was equal to A or a random number that higher/lower than A with the absolute difference between 200–400 for different conditions)

## Slide 3
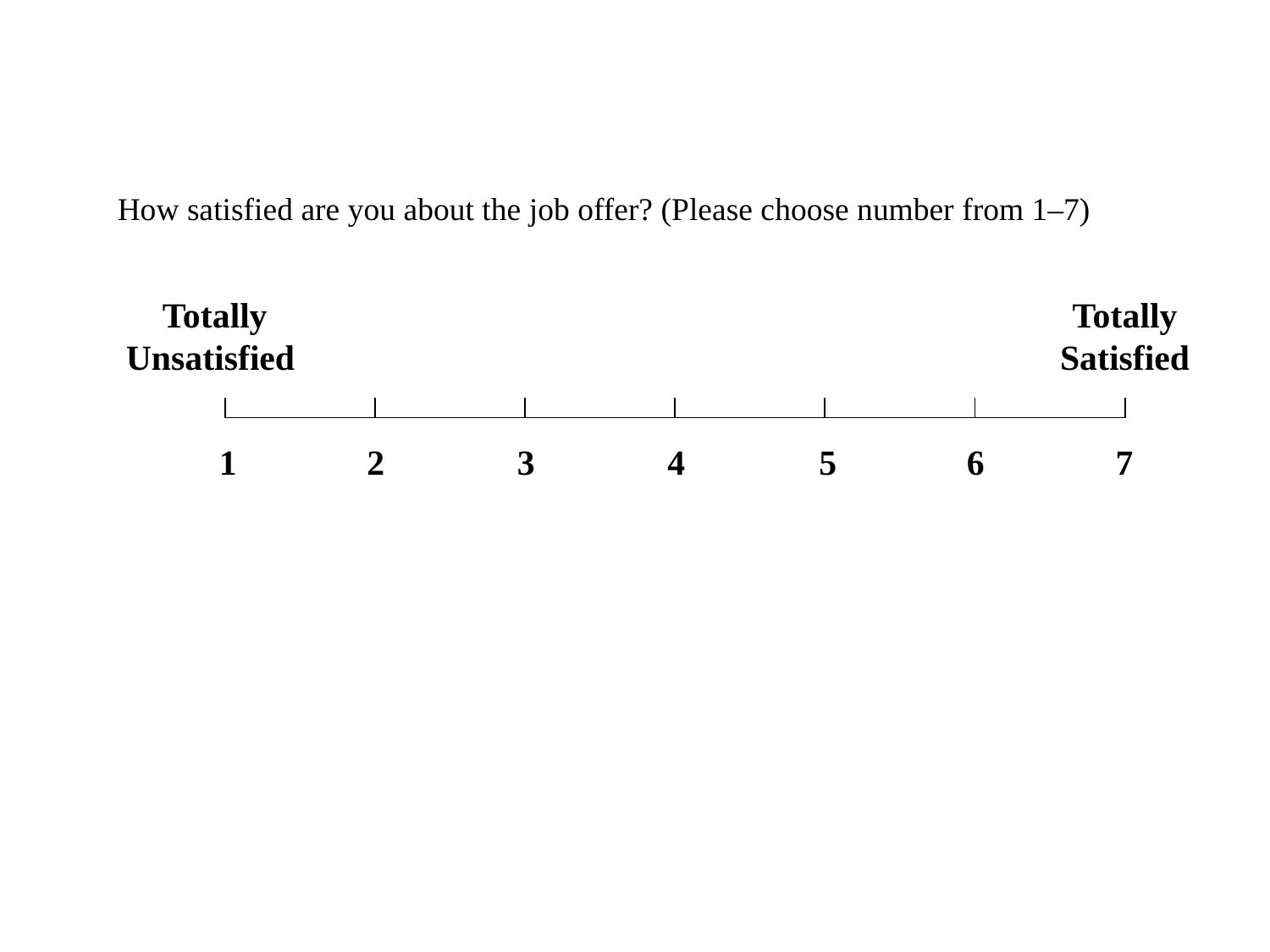

How satisfied are you about the job offer? (Please choose number from 1–7)
| | | | | | |
| --- | --- | --- | --- | --- | --- |
Totally Unsatisfied
Totally Satisfied
1
2
3
4
5
6
7
